# Designing Stable Mayonnaise: Rheological and Structural Performance Fortified with Antioxidant Star Fruit (*Averrhoa carambola*) Extract from Ultrasound-Assisted Extraction

**DOI:** 10.3390/gels12030196

**Published:** 2026-02-26

**Authors:** María Zúñiga-Navarro, Somaris E. Quintana, Luis A. García-Zapateiro

**Affiliations:** Research Group of Complex Fluid Engineering and Food Rheology, Universidad de Cartagena, Cartagena 130015, Colombia; mzunigan@unicartagena.edu.co (M.Z.-N.); squintanam@unicartagena.edu.co (S.E.Q.)

**Keywords:** mayonnaise emulsion, *Averrhoa carambola* (star fruit), phenolic compounds, antioxidant activity, rheological properties, microstructure, ultrasound-assisted extraction (UAE)

## Abstract

The preparation and characterization of phenolic extracts from *Averrhoa carambola* were performed to develop enriched mayonnaise-type emulsions, evaluating the effect on their physicochemical, rheological, and microstructural properties. Extracts were obtained by Ultrasound-Assisted Extraction (UAE) employing different ethanol:water ratios, followed by the analysis of their Total Phenolic Content (TPC) and Antioxidant Activity (AA). The 50:50 extract (AEt50) exhibiting the highest bioactivity was selected for the development of enriched mayonnaise, which was then subjected to stability, physicochemical, rheological, and microstructural analyses. Extraction yields ranged from 13% to 28%, with TPC values spanning 3251 to 4661 mg GAE/g of extract, and AA values between 49.25 and 81.67 µMol Trolox/g of extract. Subsequently, the strategic incorporation of the extract, coupled with pH adjustment, successfully maintained the pH of the final products at approximately 4.63 and preserved emulsion stability. This process resulted in a significant, dose-dependent increase in TPC and AA in the mayonnaise, with the highest concentration achieving nearly 9.0 mg GAE/g and the antioxidant activity de 60.0 μMol Trolox/g. The microstructural integrity was maintained, with all droplet sizes remaining under 4 µm, though a visible change in color (ΔE) was observed. All samples exhibited shear-thinning, non-Newtonian behavior, accurately fitted to the Ostwald–de Waele model (R^2^ > 0.982), and demonstrated a dominant elastic structure (G′ > G″) characteristic of high-quality solid-like gels. Thus, the incorporation of *Averrhoa carambola* extracts is a technically viable and effective alternative to develop stable food products enriched with functional bioactive compounds.

## 1. Introduction

Mayonnaise is a highly popular and widely consumed oil-in-water (O/W) emulsion globally, typically formulated with a high content of vegetable oil (ranging between 65 and 85%), egg, vinegar, sugar, and spices. While a staple condiment, the continuous and excessive consumption of this product has raised public health concerns, being associated with conditions such as hypertension, obesity, and cardiovascular disease [1,2]. Beyond the dietary challenges, the product’s high oil concentration renders it extremely susceptible to lipid oxidation, a process that directly compromises its organoleptic quality and shortens its shelf life. Oxidation in emulsions can be accelerated compared to bulk oils, primarily due to the increased interaction between pro-oxidants and lipids at the large oil–water interfacial area. Consequently, the use of synthetic antioxidant additives (such as BHT or BHA) is a long-standing and widespread industrial practice to maintain quality and stability [3,4].

Current consumer demand shows a strong and sustained trend towards natural and functional food products, prompting the food industry to seek cleaner label solutions. In response, natural phenolic extracts, recognized for their potent antioxidant properties, represent a clean and promising alternative to replace or significantly reduce the reliance on synthetic compounds [5]. However, integrating these natural ingredients into complex food matrices like mayonnaise presents several critical formulation challenges. Their addition can compromise the physical stability of the emulsion [6] and may negatively impact essential sensory properties (taste, aroma, creaminess) and rheological parameters, all of which are crucial for consumer acceptance [7,8]. A major technical hurdle is that many natural polyphenols exhibit limited water solubility and an often unpleasant, astringent taste, thereby requiring encapsulation or taste-masking technologies prior to direct food inclusion [9]. Therefore, research is essential to validate the functional efficacy, long-term stability, and sensory acceptability of these extracts as food additives.

Several recent studies have focused on the incorporation of natural ingredients into mayonnaise for functional enhancement. For instance, Mistrianu et al. [10] utilized beetroot peels as an antioxidant source; Ünver and Çelik [11] evaluated microcrystalline cellulose derived from almond shells; and Shaygannia et al. [12] used encapsulated phenolic compounds from lemon waste. This active research area underscores the industrial interest in the valorization of natural byproducts and underutilized resources for food innovation.

The present study focuses on *Averrhoa carambola*, or star fruit, an evergreen tree whose fleshy, angular fruit ranges from 5 to 15 cm in length [13]. The fruit is valued not only for direct consumption and processing into jams and juices [14] but particularly for its rich composition of bioactive compounds, including saponins, alkaloids, flavonoids, tannins [15], proanthocyanidins, epicatechin, and ascorbic acid [16]. These molecules confer significant biological activities, such as anti-inflammatory, antioxidant, antimicrobial, and antiulcer properties [17]. Despite this robust phytochemical profile and the ready availability of the raw material, the application of *Averrhoa carambola* extracts to fortify complex food emulsions like mayonnaise and a detailed assessment of its impact on the resulting structural, rheological, and oxidative stability remain largely unexplored in the literature.

To efficiently and sustainably harvest these valuable bioactive compounds, Ultrasound-Assisted Extraction (UAE) was employed. UAE is a recognized “green extraction” technology that utilizes acoustic cavitation to enhance mass transfer rates. This method allows for the extraction of phenolic compounds in shorter processing times, with reduced solvent consumption, and at lower temperatures compared to conventional methods. The advantages of UAE—high efficiency, cost-effectiveness, and minimal thermal degradation of sensitive compounds—make it an ideal choice for obtaining high-quality extracts with maximum antioxidant activity for food applications [18,19,20]. Therefore, the objective of this study was two-fold: first, to optimize the obtaining of phenolic extract from *Averrhoa carambola* using Ultrasound-Assisted Extraction; and second, to evaluate the application of the resulting extract for the development of an enriched mayonnaise-type emulsion, conducting a comprehensive analysis of its impact on the physicochemical properties, oxidative stability, and the final rheological and microstructural characteristics of the food matrix.

## 2. Results and Discussion

### 2.1. Characterization of Averrhoa carambola Extracts: Yields, TPC, and Antioxidant Activity

Three extracts of *Averrhoa carambola* were successfully obtained using three different hydroalcoholic solvent ratios: 25:75 ethanol:water (AEt25), 50:50 (AEt50), and 75:25 (AEt75). The influence of the solvent on the extraction yield, Total Phenolic Content (TPC), and Antioxidant Activity (AA) is summarized in Table 1.

#### 2.1.1. Extraction Yield

The extraction efficiency varied significantly (*p* < 0.05) based on the solvent’s polarity. AEt50 exhibited the highest yield with a value of 28.02 ± 8.48%, representing a notable 2.15-fold increase compared to both AEt25 (13.01 ± 1.41) and AEt75 (13.04 ± 3.54%). This outcome indicates that a hydroalcoholic solvent mixture of intermediate polarity (50:50) was optimal for solubilizing the widest range of extractable compounds from the *Averrhoa carambola* matrix, including both highly polar (e.g., sugars, organic acids) and semi-polar (e.g., certain phenolics) components. The significantly lower yields observed at the extremes (AEt25 and AEt75) suggest that a lack of balance between water and ethanol limits the overall recovery of the biomass.

#### 2.1.2. Total Phenolic Content (TPC) and Antioxidant Activity (AA)

In the same manner as the extraction yield, the TPC of the *Averrhoa carambola* extracts was highest in AEt50 (4661.14 ± 15.51 mg of GAE/g of extract), followed by AEt25 (3778.10 ± 7.75 mg of GAE/g extract) and AEt75 (3251.56 ± 64.16 mg of GAE/g extract) (Table 1). These values significantly exceeded those previously reported in the literature for similar materials, such as ethanolic extracts of Malaysian *Averrhoa carambola* L. (97.16 ± 4.29 mg GAE/g of extract) [21], hydroalcoholic (50% ethanol) extracts of Indian *Averrhoa carambola* L. (54.45 ± 0.43 mg GAE/100 g of fruit) [22], a microwave-assisted ethanolic extract with 75.57 ± 0.31 mg GAE/g of extract [23], and the ion precipitation method and macroporous resin purified carambola polyphenolic substances, which produced 436.97 ± 1.31 mg GAE/100 g [24]. The superior TPC observed in the present study can be attributed to several factors: (1) variations in fruit variety, maturity stage, and storage conditions; (2) the high concentration effect achieved by expressing the TPC per gram of dry extract rather than per gram of raw fruit; and most importantly, (3) the efficient and enhanced mass transfer provided by the Ultrasound-Assisted Extraction (UAE) method used. UAE causes the implosion of solvent cavitation bubbles, leading to the disruption of the vegetal cell membranes; this action facilitates the penetration of solvent into the cells, thereby improving mass transfer and increasing the release of bioactive compounds [20]. Specifically, the intermediate polarity of the 50:50 solvent is known to be effective for extracting a diverse range of phenolic compounds, including flavonoids, which typically exhibit moderate polarity [25].

The radical scavenging capacity (AA), measured via the ABTS+ assay, demonstrated a behavior highly correlated with the TPC, highlighting that phenolics are the principal contributors to functional activity [26]. AEt50 presented the highest AA (81.67 ± 0.95 μMol Trolox/g), followed by AEt25 (69.94 ± 1.64 μMol Trolox/g) and AEt75 (49.25 ± 2.19 μMol Trolox/g). The antioxidant mechanism of phenolic compounds is primarily associated with the donation of hydrogen atoms from their hydroxyl (OH) groups to reactive oxygen or nitrogen species, thereby terminating the radical generation cycle.

#### 2.1.3. Identification of Volatile Bioactive Compounds

Based on the superior results obtained for yield, TPC, and AA, the AEt50 extract was selected for subsequent incorporation into the mayonnaise emulsion. GC-MS analysis of the AEt50 extract revealed the presence of several bioactive compounds, including methoxyphenyloxime, hexadecenoic acid, methyl ester, 9,12-octadecadienoic acid, methyl ester; 9-octadecanoic acid, methyl ester; and octadecanoic acid, methyl ester. These compounds, particularly the fatty acid methyl esters, are recognized for their potential functional properties, including reported health benefits [27] and various biological activities [28]. The presence of these compounds confirms the extract’s potential as a valuable natural ingredient, projecting interest for its antioxidant, anti-inflammatory, antimicrobial, hypoglycemic, and hypocholesterolemic activities in food systems [29].

### 2.2. Mayonnaise Emulsions Enriched with Averrhoa carambola Extract

#### 2.2.1. Physicochemical Properties

The incorporation of natural phenolic extracts into food emulsions presents challenges, primarily due to issues related to limited water solubility and unpleasant off-tastes [9], which can compromise stability and sensory quality. During the initial formulation step, the direct addition of the *Averrhoa carambola* extract (AEt50) resulted in a significant drop in the pH of the emulsion premix, associated with a low pH of the extract (2.62 ± 0.02), leading to immediate phase separation [30]. This instability necessitated a pH adjustment by modifying the amounts of acetic acid and sodium citrate to maintain a suitable pH environment for emulsion formation. Then, the pH of the premix was adjusted to 4.6; after that, the oil phase was added and homogenized. Four stable AEt50-enriched mayonnaise emulsions were successfully prepared: a Control (0% extract), Sample-0.1 (0.1% extract), Sample-0.2 (0.2% extract), Sample-0.3 (0.3% extract), and Sample-0.4 (0.4% extract). All five samples were characterized by homogeneous physical appearances and demonstrated excellent storage stability for the duration of the 10-day study, both under refrigerated (4 °C) and ambient (25 °C) conditions. This inherent stability, even with added compounds, is attributed to the effective high-shear homogenization process, which ensures a sufficient reduction in oil droplet size, thus promoting the steric stabilization provided by the egg proteins and xanthan gum.

**Figure 1:**
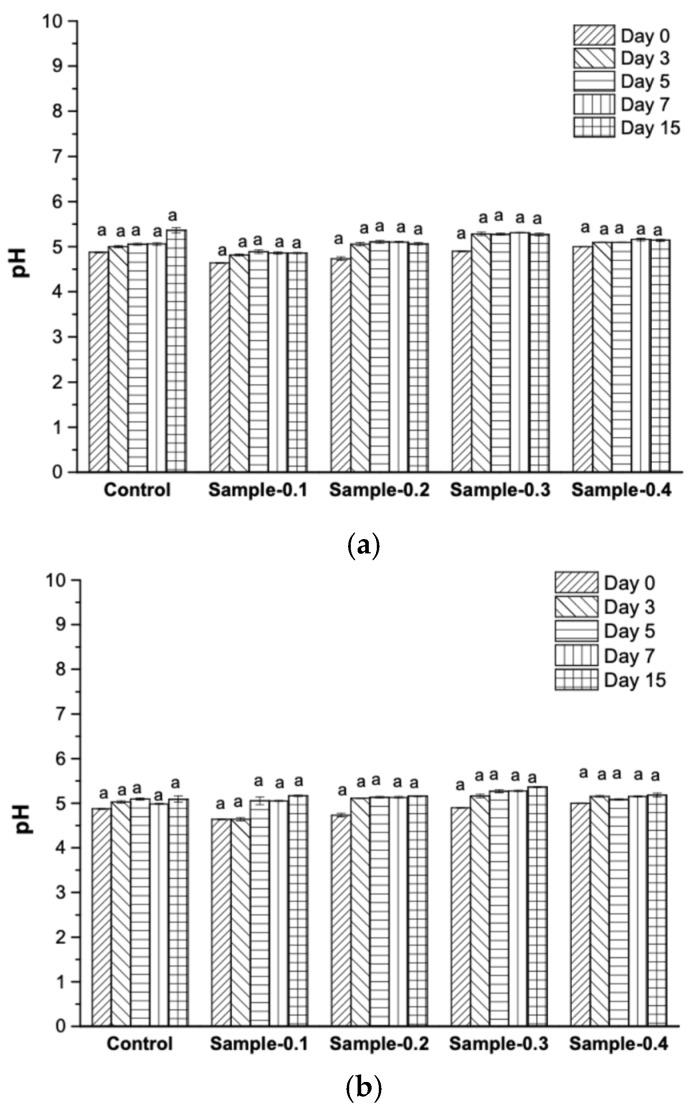
pH analysis of mayonnaises. (**a**) Samples stored at 5 °C and (**b**) samples stored at 25 °C. Different letters in the bars express statistically significant differences (*p* < 0.05).

The pH of all formulations was successfully adjusted to a near value of 4.87 ± 0.01, 4.67 ± 0.01, 4.74 ± 0.01, 4.97 ± 0.01, 4.92 ± 0.01, and 5.01 ± 0.01 for the Control, Sample-0.1, Sample-0.2, Sample-0.3, and Sample-0.4, respectively, showing no statistically significant differences across the samples (*p* > 0.05). This pH control is critical, as mayonnaise’s stability and viscoelasticity are maximized near the isoelectric point of egg yolk proteins [31]. The initial necessity for pH modification confirms the acidic nature of the *Averrhoa carambola* extract. The evolution of mayonnaise during the storage time (Figure 1) revealed that the pH of the samples was quite similar between the control and the samples with extracts, although the study was carried out under different temperature conditions, and no significant variations in pH were observed on the measurement days (*p* > 0.05), confirming its stability during storage time.

Regarding acidity (expressed as a percentage), the Control sample (0.40 ± 0.42) was found to be significantly higher (*p* < 0.05) than all enriched samples, which ranged from 0.20% to 0.27% (Table 2). This trend indicates that the pH adjustment strategy—which required modifying the quantity of acetic acid and sodium citrate to counter the acidity of the added extract—resulted in a final product with a lower titratable acidity in the enriched batches, despite the constant final pH. This behavior contrasts with some literature, such as Izidoro et al. [32], but highlights the importance of formulation fine-tuning to manage the impact of novel ingredients.

The Total Phenolic Content (TPC) and Antioxidant Activity (AA) of the mayonnaise emulsions were directly proportional to the concentration of the incorporated *Averrhoa carambola* extract, demonstrating a clear dose-dependent functional enrichment. Both TPC and AA levels in all enriched samples were significantly higher (*p* < 0.05) compared to the Control sample (TPC: 2.88 ± 0.90 mg GAE/g; AA: 32.0 ± 0.01 μMol Trolox/g of mayonnaise. The bioactive content increased in the following order: Control < Sample-0.1 < Sample-0.2 < Sample-0.3 < Sample-0.4. The highest level of fortification was achieved in Sample-0.4, which reached a TPC of 8.96 ± 2.93 mg GAE/g and an AA of 60.0 ± 0.01 μMol Trolox/g. This confirms the successful transfer of functional properties from the extract to the food matrix. This successful enrichment aligns with similar efforts to create functional emulsions using natural compounds, as reported by Mistrianu et al. [10], Raikos et al. [33] and Roman et al. [34].

#### 2.2.2. Color Properties

The addition of *Averrhoa carambola* extract significantly affected the color parameters and the microstructure of the mayonnaise emulsions. The results for the CIELAB coordinates, derived indices, and droplet size are detailed in Table 3.

The color coordinates L* (lightness), A* (red–green), and B* (yellow–blue) all showed statistically significant changes upon extract incorporation (*p* < 0.05). The lightness (L*) increased significantly when comparing the Control (68.73 ± 1.28) to all enriched samples (ranging from 72.80 to 74.90), suggesting that the extract contributed to an overall higher reflectance and scattering of light within the emulsion. However, among the enriched samples, L* tended to decrease slightly as the extract concentration increased (Table 3), indicating that higher levels of the colored phenolic compounds eventually start to absorb more light. Regarding the A* coordinate, all enriched samples showed low but significantly increased positive values, ranging from 1.03 to 1.40, compared to the Control (0.66 ± 0.11). This demonstrates a dose-dependent shift toward the red/less green region as the concentration of *Averrhoa carambola* extract increases. Similarly, the B* coordinate (yellowness) also increased significantly in all enriched samples (ranging from 12.40 to 13.20) relative to the Control (10.26 ± 1.20), with the B* value continuing to increase slightly and consistently with higher extract concentrations, confirming that the extract imparted a more pronounced yellowish hue to the final product. The Whiteness Index (WI) was significantly affected by extract addition, with the Control sample having a WI of 66.77 ± 1.17. All enriched samples exhibited significantly higher WI values (*p* < 0.05), corroborating the initial increase in L* and overall visual lightness. Finally, the total color difference (ΔE) exhibited a linear increase with the concentration of the extract (y = 9.21X + 6.585, R^2^ = 0.95). ΔE values ranged from 7.27 (Sample-0.1) to 10.06 (Sample-0.4). Since ΔE values above sim 3 are typically noticeable to the human eye, this significant difference confirms that enriching mayonnaise with plant extracts caused a marked and visually perceptible color change in the final products.

#### 2.2.3. Rheological Properties

**Figure 2:**
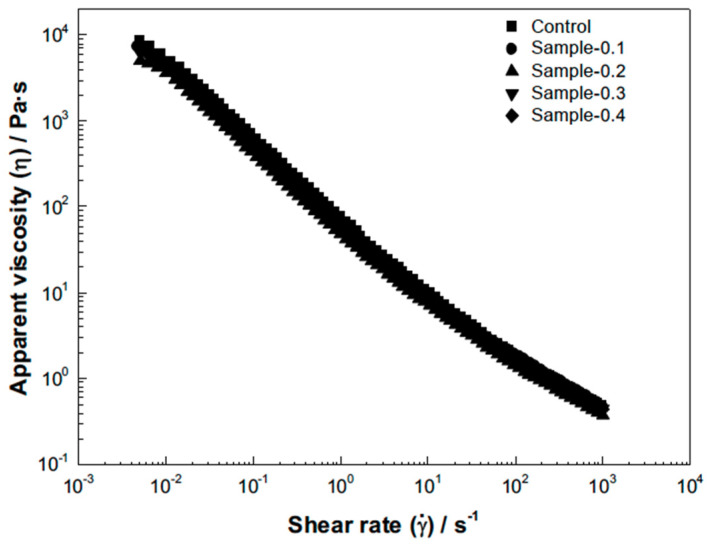
The viscous flow of samples.

Figure 2 presents the experimental data for the apparent viscosity (η) as a function of the shear rate ($\dot{\gamma }$). In all mayonnaise samples, the apparent viscosity decreased significantly with the increase in the shear rate (Figure 2), which is characteristic of non-Newtonian shear-thinning behavior [35].

Given this viscous behavior, the experimental data were successfully fitted to the Ostwald–de Waele model (Equation (3)). The high coefficient of determination (R^2^), which was greater than 0.982 in all cases (Table 4), confirms that the model accurately describes the flow behavior of the emulsions. The incorporation of the *Averrhoa carambola* extract had a measurable impact on the model parameters. The Consistency Index (k) for the Control sample exhibited the highest value. The incorporation of the extract at low concentrations initially decreased the consistency index compared to the Control, suggesting a slight reduction in overall stiffness. However, k then increased progressively with the percentage of extract added (Table 4), indicating that at higher concentrations, the dispersed phenolic compounds contribute to the total internal structure and resistance to flow. The Flow Behavior Index (n) was less than one (n < 1) in all formulations, which mathematically confirms the observed shear-thinning behavior. Furthermore, the value of n increased slightly with the concentration of the extract added, indicating that the samples became marginally closer to Newtonian behavior (where n = 1) as the extract percentage increased. These findings are consistent with results obtained by Izidoro et al. [32], who observed that banana pulp influenced the viscosity of mayonnaise-type emulsions, causing the apparent viscosity to decrease with increasing temperature and shear rate. This typical rheological behavior in complex emulsion systems—where viscosity is reduced by applied strain—is explained by the structural rupture of molecular aggregates caused by hydrodynamic forces and the increased alignment of the constituent molecules (oil droplets and hydrocolloids) along the direction of the shear [32].

The viscosity properties of mayonnaise are strictly related to the close packing of dispersed oil droplets and their interactions within the matrix, where higher viscosity is a consequence of the increased intensity of the drop–droplet interaction [36]. Table 4 summarizes the parameters obtained from fitting the experimental data to the Ostwald–de Waele model. The Consistency Index k exhibited a complex trend: its initial incorporation (Sample-0.1 and Sample-0.2) significantly decreased k relative to the Control 80.69 s^n^, suggesting an initial weakening of the internal structure. However, at the highest concentration (Sample-0.4), k reached its maximum value 88.06 s^n^, indicating that the high phenolic content eventually contributes to a robust internal resistance to flow, surpassing that of the Control. The Flow Behavior Index n was less than one in all cases, confirming the dominant shear-thinning behavior observed. The n value tended to increase with lower extract concentrations (higher for Sample-0.1), suggesting these samples are slightly closer to Newtonian fluid behavior, but decreased again at the highest concentrations.

**Figure 3:**
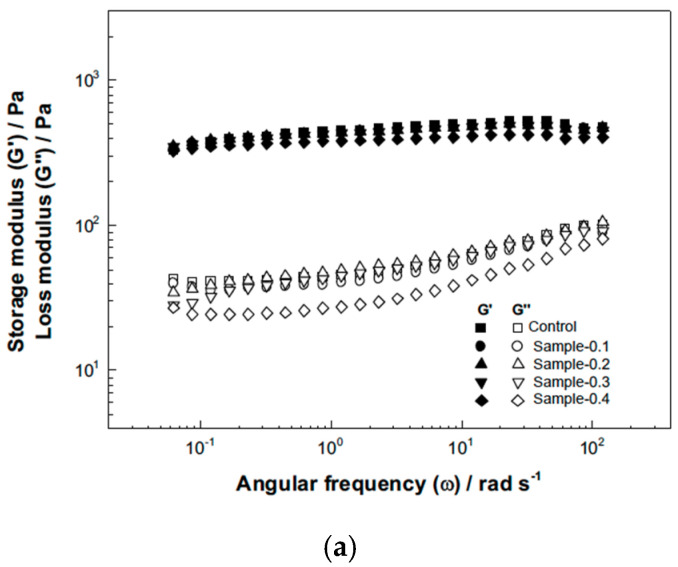

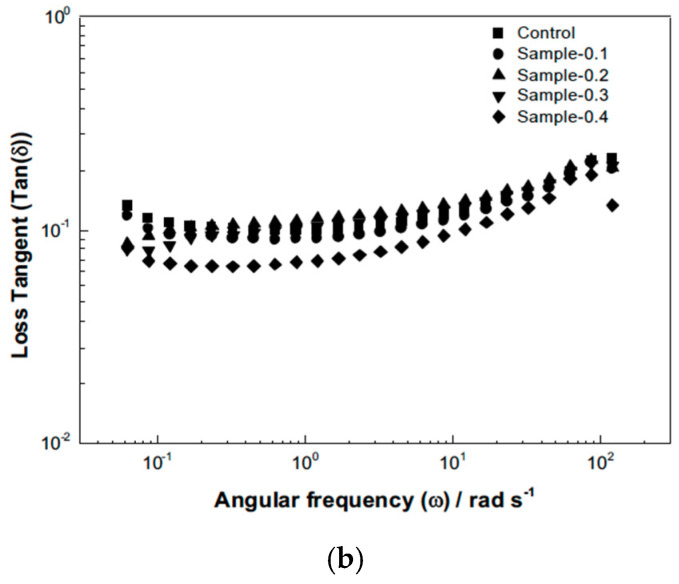
Frequency sweep (**a**) and loss tangent (**b**) of samples.

The viscoelastic properties of the mayonnaise emulsions enriched with *Averrhoa carambola* extract were evaluated using frequency sweep tests performed within the Linear Viscoelastic Region (LVR) and Viscoelastic Moduli (G′ and G″). As shown in Figure 3a, the storage modulus (G′) was consistently greater than the loss modulus (G″) across the entire angular frequency range, and both moduli exhibited a slight frequency dependence (noticeable plateau behavior). This characteristic confirms a solid-like gel structure, which is attributed to the strong network formed by the egg yolk lipoproteins absorbed at the oil droplet interfaces [37]. Table 5 provides the quantitative data for the moduli at three distinct frequencies. The moduli generally decreased with the incorporation of the extract up to the 0.4% concentration (Sample-0.4), which consistently presented the lowest G′ and G″ values across all frequencies (e.g., G′ at 0.6283 rad/s decreased from 435.3 Pa in the Control to 375.4 Pa in Sample-0.4). This decrease in the elastic modulus (G′) suggests that the added bioactive compounds interfered with the formation or strength of the protein/hydrocolloid network structure, resulting in a slightly less rigid mayonnaise structure compared to the Control. Conversely, the established principle that an increase in G′ indicates a more solid mayonnaise, per Ma et al. [38], suggests that the reduction observed at higher concentrations implies a slight structural weakening despite the maintained physical stability.

Figure 3b shows the loss tangent Tanδ values as a function of the angular frequency. The mayonnaise samples presented Tanδ values close to zero (Table 5), which reiterates that the solid behavior (G′) clearly predominates over the viscous behavior (G″) throughout the measured range, confirming the strong elastic structure characteristic of high-quality mayonnaise. Tanδ values varied with the addition of the extract, though without a clear correlation to the concentration percentage, and increased with the angular frequency, indicating a slight increase in the dominance of viscous behavior at higher deformation rates. Specifically, Tanδ values at the lowest frequency ranged from 0.069 to 0.109 across the samples, increasing to a range of 0.175 to 0.199 at the highest frequency. This trend, where Tanδ is close to zero, aligns with the findings of Martillanes et al. [39] on hydrocolloid-enriched emulsions, which also showed strong gel-like structures.

#### 2.2.4. Droplet Diameter and Storage Stability

**Figure 4:**
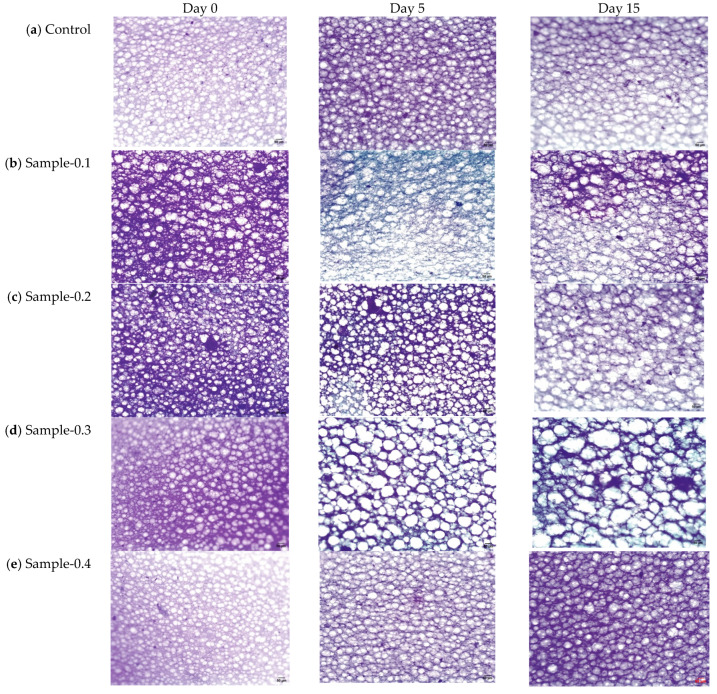
Micrograph (10×) of (**a**) Control, (**b**) Sample-0.1, (**c**) Sample-0.2, (**d**) Sample-0.3, and (**e**) Sample-0.4 stored at 5 °C. Scale bar = 50 µm.

**Figure 5:**
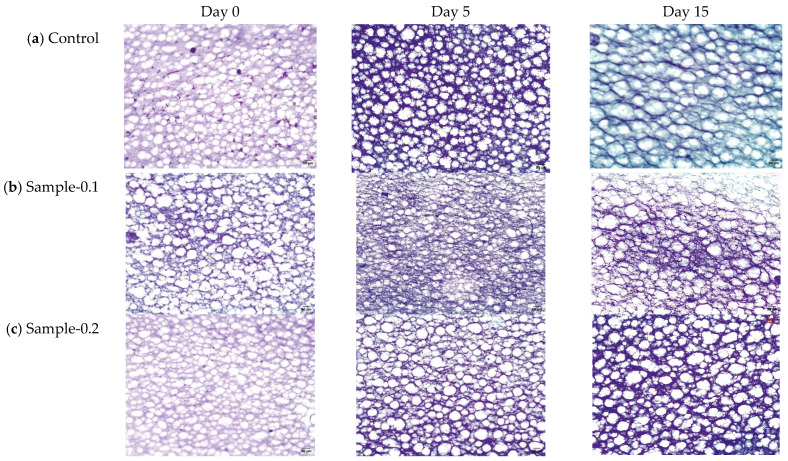

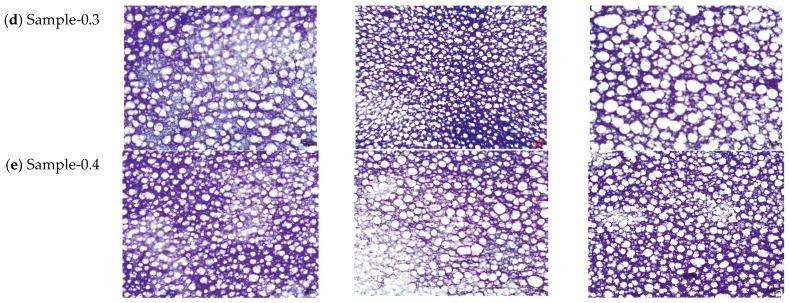
Micrograph (10×) of (**a**) Control, (**b**) Sample-0.1, (**c**) Sample-0.2, (**d**) Sample-0.3, and (**e**) Sample-0.4 stored at 25 °C. Scale bar = 50 µm.

The microstructure of the mayonnaise emulsions stored at 5 and 25 °C for 15 days was observed by microscopic imaging (Figure 4 and Figure 5). Mayonnaises presented spherical drops with polydisperse properties, showing different droplets, which is normal when handling food emulsions.

Despite the differences in droplet size, the mayonnaises showed a finely dispersed structure, and most showed a compact distribution, as is visually observed in the images (Figure 4 and Figure 5). All samples exhibited a qualitatively similar appearance, where the drops were organized in flocs, which is related to the homogenization process, which produces a reduction in the drop size [40]. When evaluating the evolution of the morphology with the storage time, a certain tendency of the drops towards flocculation was observed due to the association between the drops, which was attributed to a more attractive interaction due to van der Waals forces, hydrophobic forces, and exhaustion, which overcome repulsive interactions [40]. Then, mayonnaises with 0 and 0.1% of the extracts (Control and Sample 0.1) showed changes in droplet size over the 15 days of storage; a coalescence occurred, suggesting that the percentages of phenolic compounds added influenced the stabilization at the oil/water interface.

**Figure 6:**
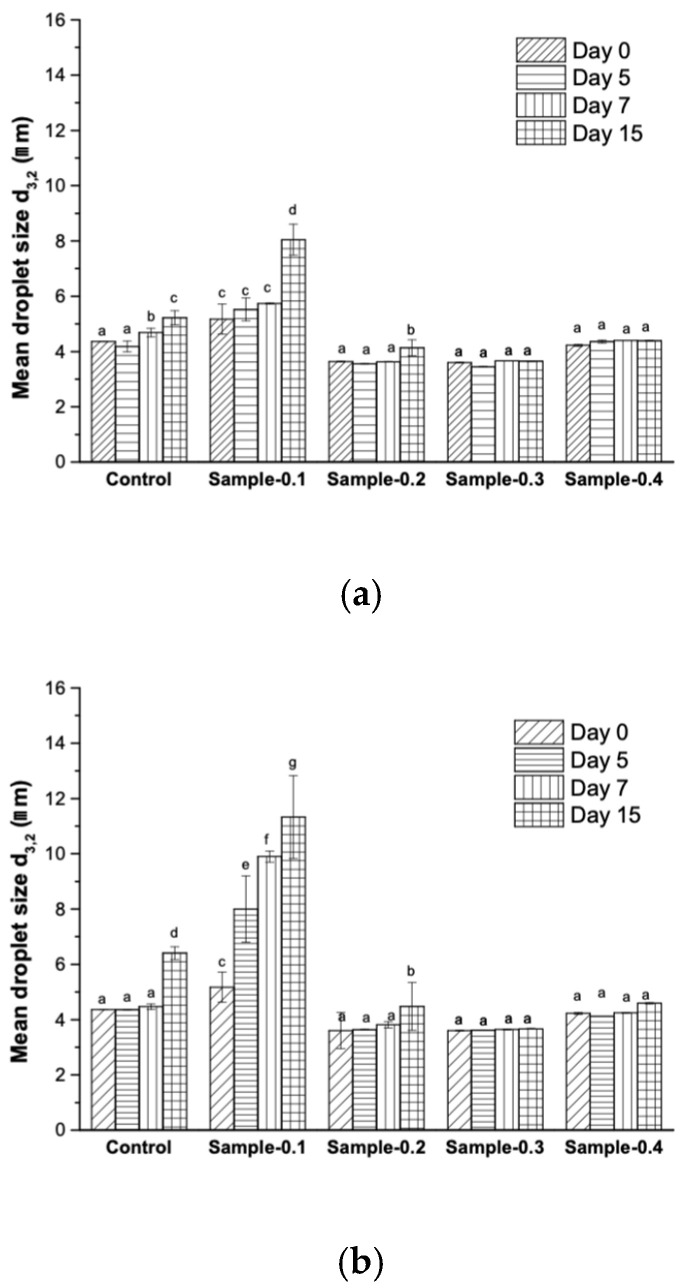
Evolution D[3;2] of mayonnaise stored at (**a**) 5 and (**b**) 25 °C. Different letters in the bars express statistically significant differences (*p* < 0.05).

The mean droplet diameters ($d_{3,2}$) of mayonnaises stored at 5 and 25 °C are shown in Figure 6 in order of micrometers. The $d_{3,2}$ of the freshy prepared mayonnaises was 4.36 ± 0.01, 5.17 ± 0.55, 3.63 ± 0.01, 3.60 ± 0.01 and 4.22 ± 0.03 for the Control, Sample-0.1, Sample-0.2, Sample-0.3 and Sample-0.4, respectively. Although the droplet size of the freshly prepared mayonnaises was small on day 0, the droplet size of the emulsions started to increase on the next day. Notably, the droplet size of Control and Sample-0.1 significantly increased (*p* < 0.05), possibly due to Ostwald ripening; meanwhile, Sample-0.2, Sample-0.3 and Sample-0.4 did not change significantly over the storage time.

Ostwald ripening and coalescence are possible destabilizing mechanisms underlying the growth in oil droplet size. Furthermore, it is evidenced that Ostwald ripening is a common phenomenon that is observed in essential oils, short-chain triglycerides, and flavor oil-in-water emulsions [41,42]. It occurs when oil molecules diffuse from small to large droplets across the aqueous phase, causing the larger droplets to consume the smaller ones [40]. It has been demonstrated that adding highly hydrophobic molecules, such as triglycerides, can control the Ostwald ripening of emulsions prepared with oils that are not highly water-soluble [43].

This observation is relevant because previous research has shown that stability and certain color attributes, like lightness (L*), are inversely related to droplet size, with higher L* values generally associated with smaller droplets due to enhanced light scattering [44]. Similarly to our findings, Martillanes et al. [39] performed a microscopic analysis on mayonnaise enriched with rice bran (*Oryza sativa* L.) and also reported that enriched samples exhibited greater homogeneity compared to the control emulsion, which had the greatest dispersion among droplet sizes. The maintenance of microstructural integrity is crucial, as the fact that the addition of the *Averrhoa carambola* extract did not significantly alter the mean droplet size—despite the potential for destabilization from the added phenolics—suggests that the pH adjustment strategy was highly effective in maintaining the emulsifying capacity of the egg yolk proteins and the structural stability of the product.

## 3. Conclusions

This study successfully demonstrated the potential of *Averrhoa carambola* extracts to enrich and enhance mayonnaise-type emulsions using Ultrasound-Assisted Extraction (UAE) and subsequent emulsification. The UAE method yielded efficient extracts, with extraction yields exceeding 13%, achieving a Total Phenolic Content (TPC) between 3251 and 4661 mg GAE/g of extract, and corresponding Antioxidant Activity (AA) between 49 and 81 μMol Trolox/g of extract. The 50:50 ethanol:water ratio (AEt50) was identified as optimal, maximizing both yield and bioactive content. The subsequent incorporation of the AEt50 extract into the mayonnaise emulsions, managed through a crucial pH adjustment using acetic acid and sodium citrate, led to the acquisition of stable microstructural products. All final emulsions maintained similar pH values (between 4.67 and 5.01) and preserved excellent physical stability over the storage period, with only a slight variation in titratable acidity observed due to the formulation adjustments. The addition of the extract successfully translated to a significant, dose-dependent increase in Total Phenolic Compounds and Antioxidant Activity within the final mayonnaise matrix, thereby creating a functional food source. Structurally, the mean droplet size showed only a slight, non-significant variation, confirming the integrity of the emulsification system, although the extract did cause a slight but visible modification in color (ΔE) and Whiteness Index (WI), which exhibited a linear dependence on extract concentration. From a rheological perspective, all mayonnaises exhibited classic non-Newtonian shear-thinning behavior, with the viscous flow data accurately fitted to the Ostwald–de Waele model (R^2^ > 0.982). Furthermore, the addition of phenolic extract to mayonnaise produced high-quality solid-like gels with an elastic structure incorporated by the emulsification process. The viscoelastic properties confirmed a strong solid-like gel structure across the frequency range, with the storage modulus (G′) consistently higher than the loss modulus (G″), evidenced by Tanδ values closer to zero. In conclusion, the successful maintenance of the physical structure and the significant enhancement of antioxidant capacity confirm that the incorporation of *Averrhoa carambola* extracts by emulsification and controlled pH adjustment is a viable and effective alternative for developing enriched microstructural products with enhanced health-promoting bioactive compounds.

## 4. Materials and Methods

### 4.1. Materials

Xanthan gum and citric acid were purchased from Tecnas SA (Medellín, Colombia). Lecithin was obtained from Tessin (Medellín, Colombia). The Analytical Reagents and Standards Glacial acetic acid (99.5%), hexane, and Ethanol (99.5%, analytical grade) were sourced from Panreac. The following chemicals and standards were purchased from Sigma–Aldrich: Sodium hydroxide (NaOH), Tween 80, acetic acid, phenolphthalein, sodium bicarbonate (99.5%), gallic acid (>98%), 2,2′-azino-bis (3-ethylbenzothiazoline-6-sulfonic acid) diammonium salt (ABTS, ≥95%), and Folin–Ciocalteu reagent. All other reagents used were of analytical grade, and olive oil was also used.

### 4.2. Sample Preparation

*Averrhoa carambola* (star fruit) cultivated in the Department of Bolívar (Colombia) was provided at commercial maturity for use in this study. The fresh fruit was initially washed with warm tap water and subsequently disinfected using a solution of sodium hypochlorite (100 ppm). Following cleaning, the fruit was sliced (or cross-sectioned) and dried in a tray dryer at 50 °C until a constant weight was achieved, a process which took approximately 10 h. Finally, the dried fruit was ground using a cutting–grinding head (IKA MF 10.1, Staufen, Germany) to obtain a fine powder with a controlled particle size of less than 250 μm.

### 4.3. Methods

#### 4.3.1. Preparation of *Averrhoa carambola* Extract

The phenolic extract of *Averrhoa carambola* was obtained using a modification of the methodology described by Quintana et al. [20]. The primary variable investigated was the ethanol:water ratio used as the extraction solvent, evaluating three different levels: 25:75, 50:50, and 75:25 (*v*/*v*). Ultrasound-Assisted Extraction (UAE) was performed using an ultrasound probe (Branson Digital Sonifier 550 model, Danbury, CT, USA) operating at a frequency of 60 kHz and an electric power of 240 W. All extractions were carried out using a fruit-to-solvent ratio of 1:10 (*w*/*v*), maintained at a temperature of 40 °C for a duration of 5 min. Subsequently, the resulting mixture was filtered. The liquid extract was then concentrated to dryness by vacuum evaporation using a rotary evaporator (IKA RV 8-V) with the water bath set at 45 °C and a pressure of 100 mbar. The final concentrated product was then lyophilized using a Labconco Freezone 1.5 L benchtop freeze-dry equipment to yield a dry powder extract. The finished extracts were stored at −10 °C until they were required for further analysis.

#### 4.3.2. Total Phenolic Compounds and Antioxidant Activity

The Total Phenolic Content (TPC) of the extracts was determined using the Folin–Ciocalteu spectrophotometric method as described by Singleton et al. [45]. The antioxidant capacity was assessed via the ABTS^+^ radical scavenging assay, following the methodology developed by Re et al. [46].

#### 4.3.3. GC-MS Analysis

The identification and quantification of volatile compounds in the samples exhibiting the highest total phenolic content and antioxidant activity were performed following the procedures described by Quintana et al. [47]. The analysis was conducted using a GC-MS-FID system (Agilent Technologies, Palo Alto, CA, USA) consisting of a Model 7890A Gas Chromatograph equipped with a split/splitless injector, coupled to a Flame Ionization Detector (FID) and a Model 5975C Triple-Axis Mass Spectrometer (MS). Chromatographic separation was achieved using an HP-5MS capillary column (30 m × 0.25 mm i.d., 0.25 µm film thickness). The oven temperature program was set as follows: initial temperature of 40 °C, increased to 150 °C at a rate of 3 °C/min (held for 10 min), and subsequently increased to 300 °C at 6 °C/min (held for 1 min). A 1 μL sample aliquot was injected in splitless mode, using helium (99.99%) as the carrier gas at a constant flow rate of 1 mL/min. The operating temperatures were maintained at 250 °C for the injector, 280 °C for the transfer line, 230 °C for the MS ion source, and 150 °C for the quadrupole.

#### 4.3.4. Mayonnaise Emulsion Preparation

Mayonnaise emulsions were prepared following the procedures described by Quintana et al. [48] and Gou et al. [49], with slight modifications. A total of five samples were prepared to evaluate the effect of incorporating different percentages of the *Averrhoa carambola* extract: 0% (Control Sample), 0.1% (Sample-0.1), 0.2% (Sample-0.2), 0.3% (Sample-0.3), and 0.4% (Sample-0.4), all expressed in weight percentage (wt). The preparation process began with the formation of the continuous (aqueous) phase. Distilled water (18%wt.) and xanthan gum (0.5 wt) were mixed at 25 °C for 10 min using a magnetic stirring plate (IKA^®^ C-MAG HS, hotplate stirrers, Staufen, Germany) at 1000 rpm to achieve a homogeneous solution. Subsequently, the remaining ingredients were added: Tween 80 (1.5%wt.), whole eggs (10% wt.), salt (3%wt.) and extract (0, 0.1, 0.2, 0.3, or 0.4%wt.); then, vinegar (3%) and sodium citrate (1%) were added to adjust to pH 4.6 ± 0.2; after that the olive oil (64%wt.) was added. The mixture was initially blended for 15 min before undergoing the emulsification step. Final homogenization was performed using an Ultra Turrax homogenizer (IKA digital T20 Ultra Turrax, Staufen, Germany) at 7000 rpm for 5 min. The finished samples were packaged and stored under refrigeration (5 °C) until used for analysis.

#### 4.3.5. Storage Stability Analysis

The storage stability of the prepared mayonnaise emulsions was evaluated over a period of 15 days. Samples of 25 mL each were transferred into cylindrical tubes and stored under two distinct thermal conditions: 4 °C (refrigeration) and 25 °C (ambient temperature). Stability was quantified based on the emulsification efficiency (E%), which was calculated as the ratio between the volume of the emulsified dispersed phase and the total volume of the dispersed phase initially incorporated into the formulation. Measurements were performed on two independent samples of each mayonnaise formulation, with each sample being measured in triplicate to ensure statistical reliability.

#### 4.3.6. Physicochemical Analysis

The acidity and pH of all prepared mayonnaise samples were determined following the standardized methodologies established by the AOAC International [50]. Furthermore, the color parameters—specifically luminosity (L*), the red–green coordinate (A*), and the yellow–blue coordinate (B*)—were evaluated according to the procedures described by Mieles-Gomez et al. [51]. Based on these measurements, the whiteness index (WI) and the total color variation (∆E*) were subsequently calculated using Equations (1) and (2), respectively:

(1) $$WI=\sqrt{100-\left[{\left(100-L^{*}\right)}^{2}+a^{2}+{B^{*}}^{2}\right]}$$

(2) $$∆E=\sqrt{{\left(∆L^{*}\right)}^{2}+{\left(∆A^{*}\right)}^{2}+{\left(∆B^{*}\right)}^{2}}$$

#### 4.3.7. Total Phenolic Compounds and Measuring Free Radical Scavenging

The Total Phenolic Content (TPC) of the mayonnaise emulsions was determined using the Folin–Ciocalteu spectrophotometric method [45]. The samples’ free radical scavenging activity was measured via the ABTS^+^ radical scavenging assay [46]. For this assay, 2 mL of a 1.0 mmol/L ABTS^+^ radical solution, prepared in methanol, was added to each test tube containing the sample extract. The resulting mixture was immediately blended and then incubated in the dark at 37 °C for 30 min. The reduction in absorbance for each solution was determined using UV–Vis spectrophotometry at a wavelength of 734 nm.

#### 4.3.8. Rheological Analysis

The rheological properties were analyzed following the procedures described by Mieles et al. [51], employing a controlled stress rheometer (Modular Advanced Rheometer System Haake Mars 60, Thermo Scientific, Germany). A rough parallel plate geometry with a diameter of 35 mm and a gap of 1 mm was used to minimize potential wall slip effects. Prior to each test, samples were subjected to a resting period (equilibration time) of 600 s to ensure consistent thermal and mechanical history across all samples.

The viscous flow test (flow curve) was performed at a constant temperature of 25 °C across a shear rate range extending from 10^−3^ to 10^3^ s^−1^. The resulting experimental data were subsequently fitted to the Ostwald–de Waele model (also known as the Power Law model), which is defined by Equation (3):

(3) $$\eta ={k\dot{\gamma }}^{n-1}$$

where η is the apparent viscosity (Pa·s), k is the consistency index, $\dot{\gamma }$ is the shear rate (s^−1^), and n is the flux index.

For viscoelastic analysis, stress sweeps were initially performed to determine the Linear Viscoelastic Region (LVR). This was achieved by applying an ascending series of stress values from 0.001 to 1000 Pa at a frequency of 1Hz. Frequency sweep tests were then conducted within the determined LVR, applying a stress value within that range while varying the frequency from 10^−2^ and 10^2^ rad/s.

#### 4.3.9. Microstructural Analysis

The microstructure of the mayonnaise emulsions was analyzed using a Primo Star optical microscope (Carl Zeiss Primo Star Microscopy GmbH, Jena, Germany) with a 100× magnification objective lens coupled with a DCMC310 digital camera and Scope Photo software (Version 3.1.615). Approximately 50 μL of each emulsion sample was placed on a slide to observe the internal distribution of the dispersed phase (oil droplets), following the procedures described by Quintana et al. [52].

#### 4.3.10. Droplet Size Analysis

The droplet size of the formulated mayonnaises was evaluated using a laser diffraction particle size analyzer (Mastersizer 3000, Malvern Panalytical, Malvern, UK). Droplet size estimations were stated as Sauter mean diameter ($d_{3,2}$) as indicated in Equation (3), where $n_{i}$ is the droplet diameter ($d_{i}$) [40]. Average values from at least three sample estimations were used for analysis.

(4) $$d_{3,2}=\frac{Volume}{surfacearea}==\frac{\sum_{i=1}n_{i}d_{i}^{3}}{\sum_{i=1}n_{i}d_{i}^{2}}$$

#### 4.3.11. Statistical Analysis

The results of the study were expressed as the mean ± standard deviation ($\bar{x}$ ± SD). All data obtained were subjected to Analysis of Variance (ANOVA) and Fisher’s LSD test using Statgraphics Centurion software (version 16.1). The coefficient of variation (C.V) was used to analyze the relationship between the standard deviation and the means. Statistically significant differences between the samples were determined at a significance level of (*p* < 0.05).

## Figures and Tables

**Table 1:** The extraction yield values, the total phenolic compound (TPC), and the antioxidant activity (AA) from *Averrhoa carambola* extract.

Extract of *Averrhoa carambola*	Yield %	TPC mg GAE/g	AA μMol Trolox/g
AEt25	13.01 ± 1.41 ^a^	3778.10 ± 7.75 ^a^	69.94 ± 1.64 ^a^
AEt50	28.02 ± 8.48 ^b^	4661.14 ± 15.51 ^b^	81.67 ± 0.95 ^b^
AEt75	13.04 ± 3.54 ^a^	3251.56 ± 64.16 ^c^	49.25 ± 2.19 ^c^

Data are the mean ± standard deviation. Different letters in the same column express statistically significant differences (*p* < 0.05).

**Table 2:** Physicochemical properties of Control, Sample-0.1, Sample-0.2, Sample-0.3, Sample-0.4 (% extracts), the total phenolic compound (TPC), and the antioxidant activity (AA).

Sample Code	Acidity %	TPC mg GAE/g	AA μMol Trolox/g
Control	0.40 ± 0.02 ^a^	2.88 ± 0.90 ^a^	32.0 ± 0.02 ^a^
Sample-0.1	0.20 ± 0.01 ^a^	3.90 ± 0.40 ^b^	39.0 ± 0.02 ^ab^
Sample-0.2	0.23 ± 0.00 ^b^	4.74 ± 0.45 ^bc^	40.0 ± 0.03 ^b^
Sample-0.3	0.27 ± 0.03 ^b^	4.57 ± 0.64 ^c^	50.0 ± 0.01 ^c^
Sample-0.4	0.26 ± 0.01 ^b^	8.96 ± 2.93 ^d^	60.0 ± 0.02 ^d^

Data are the mean ± standard deviation. Different letters in the same column express statistically significant differences (*p* < 0.05).

**Table 3:** Color parameters and droplet size. The color coordinates are L* (lightness), A* (red–green), and B* (yellow–blue); the Whiteness Index is (WI); and the total color difference is (ΔE).

Sample Code	L*	A*	B*	WI	ΔE
Control	68.73 ± 1.28 ^a^	0.66 ± 0.11 ^a^	10.26 ± 1.20 ^a^	66.77 ± 1.17 ^a^	--
Sample-0.1	74.33 ± 2.00 ^b^	1.13 ± 0.15 ^b^	12.53 ± 0.32 ^b^	72.35 ± 2.60 ^b^	7.27 ± 0.19 ^a^
Sample-0.2	74.90 ± 0.70 ^b^	1.03 ± 0.15 ^b^	12.40 ± 0.72 ^b^	71.97 ± 0.34 ^b^	8.69 ± 0.68 ^a^
Sample-0.3	73.00 ± 2.78 ^b^	1.26 ± 0.25 ^b^	12.86 ± 0.60 ^b^	68.23 ± 2.54 ^c^	9.53 ± 2.79 ^a^
Sample-0.4	72.80 ± 1.90 ^b^	1.40 ± 0.10 ^b^	13.20 ± 0.03 ^c^	69.72 ± 1.83 ^c^	10.06 ± 1.38 ^a^

Data are the mean ± standard deviation. Different letters in the same column express statistically significant differences (*p* < 0.05).

**Table 4:** Parameters for adjustment of the Ostwald–de Waele model.

Sample Code	k	n	R^2^
Control	80.69 ± 9.25 ^b^	0.121 ± 0.02 ^a^	0.982
Sample-0.1	58.61 ± 7.42 ^a^	0.181 ± 0.01 ^e^	0.988
Sample-0.2	60.28 ± 6.68 ^a^	0.164 ± 0.01 ^d^	0.982
Sample-0.3	66.54 ± 7.40 ^ab^	0.152 ± 0.02 ^c^	0.983
Sample-0.4	88.06 ± 7.37 ^b^	0.141 ± 0.02 ^b^	0.988

Data are the mean ± standard deviation. Different letters in the same column express statistically significant differences (*p* < 0.05).

**Table 5:** Storage modulus (G′), loss modulus (G″), and loss tangent (Tanδ).

Sample Code	ω = 0.6283			ω = 6.283			ω = 62.83		
	G′ Pa	G″ Pa	Tanδ	G′ Pa	G″ Pa	Tanδ	G′ Pa	G″ Pa	Tanδ
Control	435.3 ± 0.01 ^d^	43.42 ± 0.01 ^c^	0.099 ± 0.00 ^a^	487.5 ± 0.01 ^b^	55.26 ± 0.01 ^c^	0.113 ± 0.01 ^c^	497.9 ± 0.01 ^c^	94.28 ± 0.01 ^c^	0.189 ± 0.01 ^b^
Sample-0.1	424.8 ± 0.01 ^c^	38.57 ± 0.01 ^b^	0.090 ± 0.00 ^a^	472.8 ± 0.01 ^b^	50.07 ± 0.01 ^b^	0.106 ± 0.01 ^b^	482.9 ± 0.01 ^c^	89.29 ± 0.01 ^b^	0.184 ± 0.01 ^b^
Sample-0.2	424.6 ± 0.01 ^c^	46.51 ± 0.01 ^d^	0.109 ± 0.00 ^b^	469.4 ± 0.01 ^b^	59.89 ± 0.01 ^d^	0.127 ± 0.01 ^d^	459.5 ± 0.01 ^b^	91.56 ± 0.01 ^b^	0.199 ± 0.01 ^c^
Sample-0.3	417.0 ± 0.01 ^b^	41.66 ± 0.01 ^c^	0.099 ± 0.00 ^a^	463.0 ± 0.01 ^b^	55.25 ± 0.01 ^c^	0.119 ± 0.01 ^c^	458.5 ± 0.01 ^b^	87.14 ± 0.01 ^b^	0.190 ± 0.01 ^b^
Sample-0.4	375.4 ± 0.01 ^a^	25.94 ± 0.01 ^a^	0.069 ± 0.00 ^a^	403.7 ± 0.01 ^a^	35.65 ± 0.01 ^a^	0.088 ± 0.00 ^a^	397.4 ± 0.01 ^a^	69.55 ± 0.01 ^a^	0.175 ± 0.01 ^a^

Data are the mean ± standard deviation. Different letters in the same column express statistically significant differences (*p* < 0.05).

## Data Availability

Derived data supporting the findings of this study are available from the corresponding author, LGZ, on request.
